# MicroRNAs as Novel Regulators of Neuroinflammation

**DOI:** 10.1155/2013/172351

**Published:** 2013-07-25

**Authors:** Dominika Justyna Ksiazek-Winiarek, Magdalena Justyna Kacperska, Andrzej Glabinski

**Affiliations:** Department of Neurology, Epileptology and Stroke, Medical University of Lodz, Zeromskiego Street 113, 90-549 Lodz, Poland

## Abstract

MicroRNAs are relatively recently discovered class of small noncoding RNAs, which function as important regulators of gene expression. They fine-tune protein expression either by translational inhibition or mRNA degradation. MicroRNAs act as regulators of diverse cellular processes, such as cell differentiation, proliferation, and apoptosis. Their defective biogenesis or function has been identified in various pathological conditions, like inflammation, neurodegeneration, or autoimmunity. Multiple sclerosis is one of the predominated debilitating neurological diseases affecting mainly young adults. It is a multifactorial disorder of as yet unknown aetiology. As far, it is suggested that interplay between genetic and environmental factors is responsible for MS pathogenesis. The role of microRNAs in this pathology is now extensively studied. Here, we want to review the current knowledge of microRNAs role in multiple sclerosis.

## 1. Introduction

For a long time, the brain was considered as an immune privilege organ. This phenomenon was defined as a complete inaccessibility for the immune cells and immune mediators, mainly due to the impermeability of the blood-brain barrier (BBB) [1]. In the light of relatively recently obtained results from multiple studies, brain “immune privilege” has to be redefined. Now, it is considered that this term is mainly related to the specific BBB architecture, brain-resident cells immunoregulatory function, and their microenvironment, which results in restricted access of immune system elements to the central nervous system (CNS) [2, 3].

It has been proposed that specific morphological architecture of CNS borders is crucial for maintaining its immune privilege. The BBB and the blood-cerebrospinal fluid barrier (BCSFB), as an outer element of CNS borders, may be breached by activated immune cells. After migration through the brain barriers, immune cells target the cerebrospinal fluid-drained leptomeningeal and perivascular spaces [4]. The inner elements of the CNS border are glia limitans, built of astrocytic foot processes and parenchymal basement membrane [5]. Within the CSF-drained leptomeningeal and perivascular spaces, macrophages are present, which can act as antigen presenting cells (APCs) for the activated T cells [6]. After recognizing specific antigens, T cells become reactivated and result in accumulation of additional immune cells. In this stage, the inner barrier may be disturbed, and immune cells and various mediators act inside the brain [7]. Thus, in physiological conditions, CNS homeostasis is ensured by permission for immune cells migration through the BBB and BCSFB only to the CSF space, where in the absence of antigens, they patrol CNS barriers.

Other features related to brain immune privilege include absence of the lymphatic vessels in the parenchyma, which allow in other organs for draining antibodies and immune cells to peripheral lymph nodes, low expression of MHC class II on CNS resident cells, and deficiency of dendritic cells (DCs) in the parenchyma [8–10]. The immune privilege of the brain is also connected with specific CNS-driven mechanisms regulating T cells functions within CNS. Brain resident cells, namely, neurons and glia, may actively regulate macrophage and lymphocyte responses [11, 12]. It is important to notice that immune privilege is not applied for all brain regions. This phenomenon is restricted mainly to the parenchyma proper. Other regions of CNS, the ventricles, meninges and subarachnoid spaces, demonstrate immune reactivity similar to that seen on the periphery [13].

In pathological conditions, such immune privilege is disrupted leading to the development of inflammation and/or neurodegeneration, which are hallmarks of various CNS diseases, for example, Alzheimer's disease, Parkinson's disease, and multiple sclerosis (MS).

### 1.1. Neuroinflammation in Multiple Sclerosis

Multiple sclerosis is a chronic inflammatory, neurodegenerative disorder characterized by CNS infiltration of autoreactive immune cells, demyelination, acute astrogliosis, and axonal loss. The aetiology of MS is still not known, but it is widely appreciated that the disease is a result of complex interplay between genetic and environmental factors [14, 15]. Progression of this disorder leads to many neurological dysfunctions, such as loss of vision, loss of sensation, and problems with walking. About 80% of MS patients develop relapsing-remitting form of disease, while 10–15% presents primary progressive form. However, after about 10 years, roughly half of relapsing-remitting patients develop a secondary progressive stage of disease [16]. The presence of various forms of disease and differential immunopathology points toward the important role of various subsets of T-helper cells and their relative proportion present at the site of inflammation [17].

It was considered for a long period of time that T-helper type 1 (Th1) cells were the major effectors in MS pathophysiology. Th1 cells are characterized by the expression of the transcription factor T-bet and the IFN-*γ* production [18]. However, more recently, a new subset of T-helper cells have been identified, namely, Th17 cells. This subpopulation is characterized by expression of the retinoic acid receptor-related orphan receptor alpha and gamma t (ROR-*α* and ROR-*γ*t) and by the production of IL-17 [19]. It was reported that Th17 cells better attach to brain endothelium than Th1 cells, in part due to the presence of CD146 on their surface [20], and they are more effective in migration through the BBB, as they express high levels of CCR6 and CD6 [21]. Moreover, it was shown that IL-17 leads to the BBB breakdown. This cytokine is also a potent inducer of neutrophil infiltration to the site of inflammation [22]. Recruited neutrophils activate various enzymes such as matrix metalloproteinases (MMPs), proteases, and gelatinases participating in further BBB disruption [23, 24]. Studies conducted on experimental autoimmune encephalomyelitis (EAE), an animal model of MS, shows, however, that Th17 cells are not sufficient for disease induction. These results suggest that Th17 subset together with Th1 cells is responsible for disease development [25].

Another important subpopulation of CD4 + T cells—T-helper type 2 (Th2) cells—is also important for MS pathology, as it was reported that their response results in disease amelioration [26]. Regulatory T cells (Tregs) also fulfilled protective function, which has been manifested in the control of autoimmune diseases and prevention of their progression. However, in multiple sclerosis, the function but not their frequency is impaired, leading to disease progression [27]. CD8 + T cells are also implicated in MS pathology, as the clonal and oligoclonal expansion of myelin antigen-reactive CD8 + subset was observed within MS plaques [28].

Activated T cells express on their surface high levels of molecules, like very late antigen-4 (VLA-4) and leukocyte-function-associated antigen-1 (LFA-1), which has improved their adhesion to the brain endothelium and subsequent migration across BBB [29–31]. After such migration T cells undergo antigen restimulation, resulting in their accumulation and proliferation. Reactivated T cells release proinflammatory molecules, which CNS resident cells, macrophages, and B cells [32, 33]. B cells and plasma cells contribute to MS pathology, as they were detected in brain and CSF of MS patients. What is more, antibodies directed against myelin antigens have been reported in the serum of MS patients [34–36].

Microglia are the resident macrophages of the CNS. In physiological conditions, they display a quiescent phenotype that is characterized by a CD45 phenotype and lowered expression of MHC class II, B 7.2, and CD40 [37]. In stress condition they undergo morphological changes, develop phagocytic abilities, and upregulate MHC class II, B7.2, and CD40 expression becoming highly activated [37–39]. Microglia play important role in response to pathological stimuli affecting CNS, as it was shown that the overproduction of their secreted factors, such as TNF-*α*, contributed to the development and progression of MS [40].

Astrocytes, together with microglia cells, participate in innate inflammatory responses in CNS. Astrocytes react to pathogen/danger signals by cytoskeletal rearrangements associated with an increase in glial fibrillary acidic protein (GFAP) and process extension, which are the hallmark of a reactive astrogliosis, process seen in MS patients [41, 42]. They secrete interferons, thought to be crucial in the CNS defense mechanism against diverse inflammatory factors. However, prolonged unopposed proinflammatory cytokine signaling could have harmful consequences leading to pathological inflammation and neurodegeneration. Recruitment of MyD88 to the toll-IL-1 receptor (TIR) domain of the IL-1 receptor is essential in the cell signaling pathways underlying astrocyte-mediated inflammation and neurotoxicity [41, 42]. Macrophages are the major MHC class II positive cells. They have integral role in disease initiation in EAE. However, in MS pathology, they are not the only class II positive cells as the monocytes, DCs, microglia, and astrocytes could also act as an antigen presenting cells [43]. 

Members of the toll-like receptor (TLR) family are thought to be the primary evolutionarily conserved sensors of pathogen-associated molecular patterns [44]. Binding of the appropriate ligand to TLRs initiates molecular cascade leading to phagocytosis, production of a variety of cytokines, and subsequently regulation of inflammatory reaction and adaptive immune response [45]. In neuroinflammation, TLR activation may modulate the production of inflammatory cytokines [46]. The increase in TLRs expression was observed in MS brain lesions, CSF mononuclear cells, and also EAE [47, 48].

### 1.2. Biogenesis and Function of MicroRNAs

MicroRNA (miR) is a relatively novel class of small noncoding RNA, demonstrating regulatory function to mRNA translation. MiRs are approximately 22 nt long single-stranded molecules, encoded in intergenic regions, introns, exons, exon overlaps, or UTR regions [49]. They may be present as single genes, or they are arranged in clusters [50]. MiRs may be expressed as independent genes with their own transcriptional regulatory elements or from intronic sequences of protein-coding genes [50]. The presence of miR clusters may be evidence of their structural or functional (targeting mRNAs of proteins involved in the same cellular pathway) similarity between encoded miRs [51]. Most of microRNAs are transcribed by the RNA polymerase II [52], whereas some of them are results of RNA polymerase III activity [53]. They are usually transcribed as a primary transcript (pri-miRNA), which is usually several kilobases long, and contain stem-loop structures [52]. Pri-miRNA is processed in the nucleus by the microprocessor complex composed of a processing enzyme Drosha and RNA binding protein, DGCR8/Pasha [54]. This enzymatic complex performs asymmetric cleavage which generate about 70 nt long pre-miRNA containing a two nt 3′ overhang [55], essential for nuclear export [56, 57]. Pre-miRNA is transported to the cytoplasm by exportin 5 and Ran GTPase for final processing by the RNAse III enzyme Dicer, specialized to bind RNA ends, especially with short 3′ overhangs. Dicer release an approximately 22 nt double-stranded miR with a 5′ phosphate end [58]. Next, duplex RNA is incorporated into a protein complex named RNA-induced silencing complex (RISC), unwound by a helicase and separated to two ssRNAs [59]. The key protein players of RISC are RNA binding protein Argonaute (Ago) and its RNA binding partner, TRBP. The guide strand is thermodynamically favored for incorporation to the Ago complex as it has a less stable 5′ end than passenger strand, which mostly undergoes degradation [55]. 

MicroRNAs fine-tune the production of proteins within cells through repression or activation of mRNA translation [60]. They act through the interaction of their seed region mainly with the 3′ untranslated region (UTR) of the given mRNA, as it was recently shown that they can interact also with 5′ UTR or protein coding region [61, 62]. Mature miR altered mRNA expression by either inhibiting translation or signaling for mRNA degradation, depending on the degree of sequence complementarity between seed region located on the 5′ end of miR (between 2 and 8 nt) and binding site of mRNA, although sequences outside the seed region are also important for recognizing targets and optimizing mRNA regulation [63]. The seed area may be supplemented by nucleotide 8 of miR, by adenine from nucleotide 1 of miR, or by both of them. The newly discovered microRNA's seed region comprises of nucleotides 3 to 8 [64–66]. 

MiRs are universal regulators of protein expression, as a single molecule can regulate translation of hundreds of targeted mRNAs and single mRNA's 3′ UTR may have multiple binding sites for various microRNAs. MiRs may function in two ways to enhance their regulatory capacity, by targeting multiple binding sites present within 3′ UTR of mRNA or by targeting multiple genes from the same cellular pathway [67]. It is estimated that in mammals, miRs may regulate more than 60% of protein-coding genes [67]. Moreover, microRNAs may function not only in cytoplasm, as they were also identified in the nucleus [68, 69], where they may act as an epigenetic regulators of gene expression [70].

MicroRNAs play crucial role in the regulation of diverse biological processes, like tissue development and homeostasis [71], cell proliferation and differentiation, apoptosis, and immune system function [72]. They are crucial for system's ability to coping with external and internal perturbations, as they regulate the mRNA expression profile by reinforcing transcription, reducing defective and overabundant transcript copy number [67]. Altered biogenesis and/or function of miR is implicated in the various pathological processes such as autoimmunity, viral infections, neurodegeneration, and inflammation [73]. Dysregulated miRs contribute to the development of various diseases, for example, cancer, cardiovascular, or neurological diseases [71, 74, 75]. It was shown that inflammation may regulate miR biogenesis. TLR ligands, antigens, or cytokines can alter miR expression level through specific transcription factors regulation [76–78]. It was also reported that cytokines may lead to deregulation of Dicer expression resulting in aberrant pre-microRNA processing [79].

Defective miR regulation during diverse immune processes may be associated with several human diseases. There are various processes, except for the impact of inflammatory factors, contributing to such regulation such as mutations, epigenetic inactivation, or gene amplification [80].

### 1.3. The Role of miRs in Neuroinflammation and MS

In the light of rapidly accumulating data from various studies, it has been concluded that miRNAs are crucial regulators of immune cell development and function. Diverse alterations in their biogenesis and regulatory role have been observed in inflammatory diseases such as rheumatoid arthritis, psoriasis, and multiple sclerosis. As multiple sclerosis is one of the most common neurological debilitating disease of as yet unknown etiology, we want to review in this section current knowledge regarding the role of these small noncoding RNAs in the MS inflammation (Table 1).

Multiple sclerosis is considered as a T-cell-mediated disorder, so it is not surprising that researchers attention is directed toward the role of miRs deregulation in T-cell maturation, activation, and function. One of the first identified miRs related to the T cells is miR-155. Expression of this miR has been linked to T cells activation following TCR stimulation [81, 82]. Differentiation of T-helper cells is also dependent on miR-155 expression. Mice deficient in this miR have demonstrated normal lymphocyte development, but altered Th1/Th2 ratio with presence of increased Th2 polarization and elevated levels of Th2 cytokine production [83–85]. Studies conducted by Cox et al. on MS patients identified significant downregulation of hsa-miR-17 and hsa-miR-20a [86]. Using knock-in and knock-down approaches it was concluded that these two miRs participate in T-cell activation regulation. FOXO1, belonging to forkhead family transcription factors, is a suppressor of T-cell proliferation, activation, and differentiation. Downregulation of FOXO1 expression, in part by hsa-miR-182-5p, is crucial for the T-cell clonal expansion [87].

It has been suggested that miR-146a expression may play a role in cell fate determination. Studies conducted on mouse lymphocytes have shown that the level of miR-146a is increased in Th1 cells and decreased in Th2 cells, when compared to its expression in naive T cells [88]. The polarization of Th1 cells may be in part regulated also by miR-21, as IL-12p35 is one of its potential targets. IL-12p35 is a subunit of IL-12 [89], cytokine which controls Th1 differentiation and IFN-*γ* secretion by the synergistic action with IL-18 [90]. 

Du et al. indicated, in the studies conducted on MS Chinese patients, that miR-326 is a regulator of Th17 cells differentiation [91]. It was shown that *in vivo* silencing of miR-326 caused reduced number of Th17 subset and mild EAE, whereas its overexpression resulted in elevated level of Th17 cells and more severe EAE. It was concluded that miR-326 acts on Ets-1, a negative regulator of Th17 differentiation [91]. Mycko et al. reported significant upregulation of another miR, namely, miR-301a in T-helper cells in response to MOG antigen [92]. MiR-301a regulates Th17 differentiation through inhibition of PIAS3, a negative regulator of the STAT3 activation pathway [92]. Inhibition of miR-301a results also in decreased secretion of IL-17 and downregulation of ROR-*α* and ROR-*γ*t expression [92]. Moreover, IL-17A expression may be inhibited by miR-146 function [93]. O'Connell et al. have revealed in MS animal model the positive role of miR-155 in autoimmunity as this miR drives Th17 differentiation of T cells [94]. As mentioned earlier, miR-301a regulates Th17 differentiation. However, it was reported that this microRNA is also expressed due to CD8 + T cells activation, where it may function as a regulator of CD69 expression [95].

MicroRNAs play important roles in regulatory T cells (Tregs) that are important protective cells preventing development and progression of autoimmune diseases. MiR-155 was shown to regulate Treg development, as miR-155-deficient mice have reduced numbers of Tregs [96], whereas miR-146 and miR-17-92 cluster regulate Treg function [97]. MiR-146a, when highly expressed in this T cell subset, selectively controls Treg-mediated inhibition of IFN-*γ*-dependent Th1 response and inflammation by activating STAT1 expression [98]. It was also reported that in human Tregs miR-21 functions as a positive indirect regulator of Foxp3 expression, while miR-31 acts as its negative regulator [99]. Recently, it was shown that Foxp3 represses miR-142-3p expression, leading to exacerbation in cAMP production and suppressor function of Treg cells [100].

Development of bone marrow-derived B cells is partially regulated by miR-181a expression. During B-cell development from the pro-B to the pre-B-cell stage, the expression level of miR-181a decreases [101]. Upregulated expression of miR-181a in pro-B stage inhibits such stage transition. MiR-181a is also considered as a positive regulator of B cells differentiation, as its expression in hematopoietic stem and progenitor cells leads to an increase in fraction of B-lineage cells and decrease in T cells or myeloid cells [101]. Conditional deletion of Dicer in mouse B cells also results in complete B cell development blockage [102]. Similar results were obtained for miR-17-92-deficient B-cells. Inhibition of miR-17-92 expression results in elevated levels of proapoptotic protein Bim and inhibition of B cell development at the pro-B to pre-B stage [103]. MiR-150 is known for its role in B lymphocytes development. It was shown that its constitutive expression may lead to similar results as seen for Dicer- and miR-17-92-deficient mouse [104]. MiR-150 controls B-cell differentiation by targeting transcription factor—c-Myb [105]. 

As observed for the first time in T cells, miR-155 is crucial also for B-cell functions. It has been reported that miR-155 is important in B-cell responses to thymus-dependent and- independent antigens [85]. It was also shown that miR-155 regulates immunoglobulin class switching to IgG [83]. Elevated expression of PU.1, a target for miR-155, leads to the reduced production of IgG1 cells. This suggests that miR-155 regulation of PU.1 may be in part responsible for proper generation of immunoglobulin class-switched plasma cells [85]. MiR-155 also represses activation-induced cytidine deaminase, enzyme essential for immunoglobulin gene diversification [106, 107]. Moreover, miR-155-deficient B cells generated reduced extrafollicular and germinal center responses [85]. Recently, immunoglobulin class switch recombination was also connected with the function of miR-181b. Elevated expression of miR-181b results in impairment of this process [108].

MiR-223 is mainly expressed in myeloid cells and functions as a regulator of granulocytopoiesis. It was reported that miR-223 negatively regulates both the proliferation and activation of neutrophils by targeting Mef2c, a transcription factor promoting myeloid progenitor proliferation [109]. Moreover, neutrophils deficient in this miR are hypermature and hypersensitive to activating stimuli and that they display aberrant pattern of lineage-specific marker expression [109]. However, there are contradictory results from different study indicating that miR-223 is a positive regulator of granulocytopoiesis [110]. Additionally, miR-223 modulates the NF-*κ*B pathway leading to alterations in immune inflammatory responses [111]. This opposed results may reflect complex interplay between the miRNA and its target pathway. It was reported that another miR, namely, miR-9, is similarly upregulated in human peripheral monocytes and neutrophils. This upregulation is mediated by proinflammatory signals conveyed in a MyD88- and NF-*κ*B-dependent manner [112].

Results obtained from numerous studies have shown that expression of toll-like receptors (TLRs) may be regulated by miR-146a. Expression of miR-146a was significantly upregulated by TNF-*α* and IL-1*β* and blocked by its receptor antagonist. Interestingly, miR-146a acts through suppression of proinflammatory proteins such as interleukin-1 receptor-associated kinase 1/2 (IRAK1/2) and TNF receptor-associated factor (TRAF) as well as IL-1*β* in a negative feedback loop [113]. It may also directly interacts with complement factor H (CFH), a repressor of the inflammatory reaction, leading to exacerbation of inflammation [114, 115].

Ponomarev et al. provided evidence that miR-124 has crucial role in maintaining quiescent phenotype of microglia in mouse EAE—experimental model of MS [116]. Expression of miR-124 was significantly downregulated in activated microglia, resulting in subsequent upregulation of CCAAT enhancer-binding proteins (C/EBPalpha) and PU.1 expression. PU.1 plays important role in the activation of monocytic lineage phenotype [117]. During EAE, expression of brain-specific miR-124 was observed only in microglia, suggesting that this small noncoding RNA participates in the resting phenotype of these cells through the regulation of C/EBPalpha/PU.1 pathway [116]. It was shown that immune response in microglia could be modulated by miR-155. MiR-155 decreases expression level of suppressor of cytokine signaling 1 (SOCS-1) leading to elevated cytokine and NO production [118]. Recently, studies conducted by Iyer et al. reported regulatory role of miR-146a in astrocyte-mediated inflammatory response [113]. In addition, it was reported that in multiple sclerosis lesions miR-155 is highly expressed in reactive astrocytes [119]. By the application of miR-155 inhibitor oligonucleotide, Tarassishin et al. have shown that miR-155 regulates astrocyte proinflammatory gene expression [120].

It was reported by Fontana et al. that monocytopoiesis is partially controlled by three miRNAs: miR-17-5p, miR-20a, and miR-106a. These microRNAs regulate expression of transcription factor acute myeloid leukaemia-1 (AML1) [121]. However, AML1 binds to and transcriptionally inhibits expression of those three miRs in a negative feedback loop [121]. Another transcription factor related to monocyte differentiation, PU.1, activates transcription of miR-424. Upregulation of miR-424 stimulates monocyte differentiation [122]. Studies by Junker et al. conducted in active MS lesions identified three upregulated miRNAs: miR-155, miR-326, and miR-34a that target the same transcript—CD47 mRNA [119]. CD47 is a membrane glycoprotein, which mediates macrophage inhibition. The interaction of CD47 with signal regulatory protein-*α* present on macrophages inhibits IgG or complement-induced phagocytosis. Downregulation of CD47 expression results in promotion of myelin phagocytosis by macrophages during MS course [123, 124].

The regulation of miRs is seen also in dendritic cells (DCs). Deficiency in miR-155 was shown to affect their function as an APC in EAE [83]. It was also reported that miR-155 knockdown results in increase in the proinflammatory cytokine IL-1*β* expression [125]. Other miRs related to DCs are miR-34 and miR-21. They were reported to play important role in myeloid-derived DC differentiation through regulation of Jagged1 and WNT1 mRNA translation [126].

The induction of central tolerance is regulated during T-cell maturation to maintain proper immune system functioning. There is evidence for strong correlation between the sensitivity of the T cells to antigen and levels of miR-181a [127]. A decrease in TCR sensitivity may result in self-tolerance breakdown and subsequent autoimmunity development [128]. The high levels of miR-181a may contribute to the decreased activation threshold of autoreactive T cells, while inhibition of miR-181a expression in the immature T cells lowers their sensitivity. The function of miR-181a is mainly mediated by downregulation of several protein tyrosine phosphatases, such as SHP-2, DUSP5, and DUSP6 [129].

The process of immune cells recruitment into the brain parenchyma is also regulated by microRNAs. It was revealed that miR-17 and miR-126 targeted ICAM1 and VCAM1 mRNA, respectively [130, 131]. Moreover, it was shown that miR-124 and -126 have regulated expression of CCL2, a chemokine responsible for monocytes recruitment to brain parenchyma. Hence, miRNAs associated with inflammatory response may also act as a potential neuroprotectants [132, 133].

## 2. Conclusions

Inflammation is an extremely important and complex biological process of the immune system activated in response to harmful stimuli such as diverse pathogens or cell damage. Its main physiological function is manifested in removal of pathogens and damaged cells or healing process [134]. However, in some circumstances, inflammatory response may be unleashed from the biological control leading to tissue damage. Dysregulated inflammatory reaction can result in development of autoimmune disorders such as rheumatoid arthritis, psoriasis, or multiple sclerosis [135, 136]. 

 Multiple sclerosis is a multifactorial neurological disease characterized by the presence of inflammatory brain infiltrates and subsequent neurodegeneration. MS is a progressive disorder affecting mostly young adults. It is stated that MS develops in genetic susceptibility individuals, which are exposed for action of various predisposing environmental factors. Although multiple sclerosis has been studied for many years, exact factors underlying its pathogenesis remain still unknown.

 It has been recently shown that less than 2% of human genome undergoes translation into proteins. However, more than half of the human genome is transcribed, suggesting that most of the transcripts account for noncoding RNAs (ncRNAs). It has now become obvious that such RNA molecules are not the “junk sequences” as it was thought before. Rather, they demonstrate important regulatory role [137]. Noncoding RNAs may be divided into two groups: long and short ncRNAs. Within each of these groups, we can further distinguish various subtypes. Most of them have not known or only partially discovered function. One of the most extensively studied groups of ncRNAs are microRNAs. These small RNAs are crucial posttranscriptional regulators altering diverse cellular processes. It was reported that they are important fine-tuners of immune responses. Both the induction and repression of miRNA expression mediated by various inflammatory stimuli may lead to alteration in immune cells differentiation and function, thus leading to the development of neuroinflammatory, autoimmune diseases (Table 1).

Recently, researchers attention is pointed toward the function of ncRNAs as an another level of genetic regulation, which may contribute to MS pathogenesis. As it was shown in multiple studies, microRNAs play diverse roles in immune system, indicating that interplay between miRs and their targets is rather complex and multifactorial. What further complicates the issue, miRs are not functioning only inside particular cell types but also they act as a signal-carrying paracrine elements contributing to cell-cell communication [138, 139].

Further studies should be conducted to reveal the role of microRNAs and other ncRNAs as they compose complex and crucial regulatory machinery, being also potential and promising targets for novel therapies.

## Figures and Tables

**Table 1:** MicroRNA regulation of inflammatory cells differentiation and function.

Cell type	Process	MicroRNA	Notes
T cells	T-cell differentiation	miR-155	—
		miR-182-5p	Regulation of FOXO1 expression
		miR-146	High level in Th1, low level in Th2, and regulation of IL-17A expression
		miR-21	Regulation of Th1 differentiation and IFN*γ* secretion, positive regulator of Foxp3 expression
		miR-326	Th17 differentiation through regulation of Ets-1 expression
		miR-301a	Th17 differentiation through regulation of PIAS3 expression, regulation of IL-17 secretion, and ROR*α* and ROR*γ*t expression
		miR-31	Negative regulator of Foxp3 expression
	T-cell activation	miR-155	—
		miR-17	—
		miR-20a	—
		miR-182-5p	Regulation of FOXO1 expression
		miR-301a	CD8+ activation through CD69 regulation
		miR-146	Regulation of Treg function
		miR-17-92	Regulation of Treg function
		miR-142-3p	Regulation of Treg function
	Sensitivity to Ag	miR-181a	Regulation by targeting, for example, SHP-2, DUSP5, and DUSP6

B cells	Pro-B to pre-B stage transition	miR-181a	—
		miR-17-92	Antagonist of proapoptotic genes
		miR-150	Regulation of c-Myb expression
	B-cell differentiation	miR-181a	Positive regulator
	Response to Ag/Ig production	miR-155	Regulation of response to various antigens, Ig class switching to IgG, Ig gene diversification, and extrafollicular and germinal center responses
		miR-181b	Regulation of Ig class switch recombination

Granulocytes	Granulocytopoiesis	miR-223	Regulation of Mef2c expression

Microglia	Quiescent phenotype	miR-124	Regulation of CEBP*α*/PU.1 pathway
	Inflammatory response	miR-155	Regulation of SOCS-1 expression

Astrocytes	Inflammatory response	miR-146a	Negative feedback regulator
		miR-155	Regulation of proinflammatory gene expression

Monocytes	Monocytopoiesis	miR-17-5p	Regulation of AML1 expression
		miR-20a	Regulation of AML1 expression
		miR-106a	Regulation of AML1 expression
	Monocyte differentiation	miR-424	—

Macrophages	Macrophage activation	miR-155	Regulation of CD47 expression
		miR-326	Regulation of CD47 expression
		miR-34a	Regulation of CD47 expression

Dendritic cells	APC function	miR-155	—
	DC differentiation	miR-34	Regulation of Jagged1 and WNT1 expression
		miR-21	Regulation of Jagged1 and WNT1 expression

Endothelial cells	Cell migration	miR-17	Regulation of ICAM1 expression
		miR-126	Regulation of VCAM1 expression
